# Quality-of-Life and Recurrence Outcomes Following Laparoscopic Elective Sigmoid Resection vs Conservative Treatment Following Diverticulitis: Prespecified 2-Year Analysis of the LASER Randomized Clinical Trial

**DOI:** 10.1001/jamasurg.2023.0466

**Published:** 2023-04-19

**Authors:** Alexandre Santos, Panu Mentula, Tarja Pinta, Shamel Ismail, Tero Rautio, Risto Juusela, Aleksi Lähdesmäki, Tom Scheinin, Ville Sallinen

**Affiliations:** 1 Gastroenterological Surgery, University of Helsinki and Helsinki University Hospital, Helsinki, Finland; 2 Department of Surgery, Seinäjoki Central Hospital, Seinäjoki, Finland; 3 Department of Surgery, Medical Research Center, Oulu University Hospital, Oulu, Finland; 4 Department of Surgery, Vaasa Central Hospital, Vaasa, Finland; 5 Department of Surgery, Hyvinkää Hospital, Hyvinkää, Finland; 6 Transplantation and Liver Surgery, University of Helsinki and Helsinki University Hospital, Helsinki, Finland

## Abstract

**Importance:**

Both elective sigmoid resection and conservative treatment are options for patients with recurrent, complicated, or persistent painful diverticulitis; understanding outcomes following each can help inform decision-making.

**Objective:**

To compare outcomes of elective sigmoid resection and conservative treatment for patients with recurrent, complicated, or persistent painful diverticulitis at 2-year follow-up.

**Design, Setting, and Participants:**

This multicenter parallel open-label individually randomized clinical trial comparing elective sigmoid resection to conservative treatment in patients with recurrent, complicated, or persistent painful diverticulitis was carried out in 5 Finnish hospitals between September 2014 and October 2018. Follow-up up to 2 years is reported. Of 85 patients randomized and included, 75 and 70 were available for QOL outcomes at 1 year and 2 years, respectively, and 79 and 78 were available for the recurrence outcome at 1 year and 2 years, respectively. The present analysis was conducted from September 2015 to June 2022.

**Interventions:**

Laparoscopic elective sigmoid resection vs conservative treatment (patient education and fiber supplementation).

**Main Outcomes and Measures:**

Prespecified secondary outcomes included Gastrointestinal Quality of Life Index (GIQLI) score, complications, and recurrences within 2 years.

**Results:**

Ninety patients (28 male [31%]; mean [SD] age, 54.11 [11.9] years and 62 female [69%]; mean [SD] age, 57.13 [7.6] years) were randomized either to elective sigmoid resection or conservative treatment. After exclusions, 41 patients in the surgery group and 44 in the conservative group were included in the intention-to-treat analyses. Eight patients (18%) in the conservative treatment group underwent sigmoid resection within 2 years. The mean GIQLI score at 1 year was 9.51 points higher in the surgery group compared to the conservative group (mean [SD], 118.54 [17.95] vs 109.03 [19.32]; 95% CI, 0.83-18.18; *P* = .03), while the mean GIQLI score at 2 years was similar between the groups. Within 2 years, 25 of 41 patients in the conservative group (61%) had recurrent diverticulitis compared to 4 of 37 patients in the surgery group (11%). Four of 41 patients in the surgery group (10%) and 2 of 44 in the conservative group (5%) had major postoperative complications within 2 years. In per-protocol analyses, the mean (SD) GIQLI score was higher in the surgery group compared to the conservative treatment group by 11.27 points at 12 months (119.42 [17.98] vs 108.15 [19.28]; 95% CI, 2.24-20.29; *P* = .02) and 10.43 points at 24 months (117.24 [15.51] vs 106.82 [18.94]; 95% CI, 1.52-19.33; *P* = .02).

**Conclusions and Relevance:**

In this randomized clinical trial, elective sigmoid resection was effective in preventing recurrent diverticulitis and improved quality of life over conservative treatment within 2 years.

**Trial Registration:**

ClinicalTrials.gov Identifier: NCT02174926

## Key Points

**Question:**

Does sigmoid resection improve long-term quality of life of patients with recurring, complicated, or persistent painful diverticulitis compared to conservative treatment?

**Findings:**

In this prespecified 2-year follow-up of a randomized clinical trial including 85 patients in the intention-to-treat analyses, patients allocated to surgery had better outcomes at 2 years compared to patients allocated to conservative treatment.

**Meaning:**

In this study, elective sigmoid resection improved quality of life and decreased recurrence of diverticulitis in 2-year follow-up; these findings may help in decision-making regarding when to proceed to elective surgery for diverticulitis.

## Teaser

This 2-year follow-up of the LASER randomized clinical trial evaluates quality-of-life outcomes among patients with recurring, complicated, or persistent painful diverticulitis following surgery vs conservative treatment.

## Introduction

Acute diverticulitis is a common reason to seek emergency medical attention.^1^ At least two-thirds of the episodes are uncomplicated, but a subset of patients present with complicated diverticulitis.^2^ Although uncomplicated diverticulitis does not require antibiotic treatment and rarely causes significant morbidity or mortality,^3,4^ it has a tendency to recur^5,6,7^ or develop into persistent painful diverticulitis.^8^ Further, some of the episodes of complicated diverticulitis (mainly abscesses) may be treated conservatively, but about one-third recur if the affected bowel part is not surgically removed.^9,10,11^

Elective sigmoid resection has been used as a treatment method for recurring uncomplicated or persistent painful diverticulitis or as a preventive measure for recurrence after complicated diverticulitis has been treated conservatively. While historically elective sigmoid resection was advocated with a low threshold (ie, after the first instance of uncomplicated diverticulitis in young patients or the second in others), current guidelines recommend a tailored case-by-case approach without clear relative or absolute indications.^12,13,14,15^ These guidelines have relied on retrospective and nonrandomized studies as, to our knowledge, only 1 randomized clinical trial comparing elective sigmoid resection to conservative treatment has reported long-term outcomes. Patients included in the Diverticulitis Recurrences or Continuing Symptoms: Operative Versus Conservative Treatment (DIRECT) trial,^16,17^ mostly with persistent painful diverticulitis, reported increased quality of life (QOL) after surgical treatment up to 5 years from randomization in addition to a reduced rate of recurrent episodes of diverticulitis. To help fill this research gap, we commenced the Laparoscopic Elective Sigmoid Resection vs Conservative Treatment Following Diverticulitis (LASER) randomized clinical trial.

## Methods

### Study Design

The LASER trial was a multicenter parallel group open-label individually randomized clinical trial comparing elective laparoscopic sigmoid resection to conservative treatment in patients with recurrent, complicated, or persistent painful diverticulitis. The trial was carried out in Finland at 2 university hospitals (Helsinki University Hospital and Oulu University Hospital) and 3 community (central) hospitals (Etelä-Pohjanmaa Central Hospital, Vaasa Central Hospital, and Hyvinkää Hospital). The trial was approved by the ethical committee of Helsinki University Hospital and by the institutional review board at each center. Results of 6-month follow-up have been published earlier, together with detailed information of the methods,^18^ and only key methodology is summarized here. The full trial protocol can be found in Supplement 1. The Consolidated Standards of Reporting Trials (CONSORT) reporting guideline has been followed in reporting this trial.

### Participants

Patients were eligible for inclusion if they had 3 or more episodes of left colon diverticulitis within a 2-year period with at least 1 episode verified using computed tomography (CT), 1 or more episodes of conservatively treated complicated left colonic diverticulitis, or prolonged pain or disturbance in bowel habits over 3 months after an episode of CT-verified acute left colonic diverticulitis. The term *persistent painful diverticulitis* corresponds to what is now widely referred to as symptomatic uncomplicated diverticular disease (SUDD), and is defined as the presence of diverticula, symptoms of abdominal pain, bloating, and bowel habit change (such as diarrhea or constipation) without macroscopic inflammation.^19^ Patients were excluded if they had multimorbidity preventing elective surgery, contraindication to laparoscopy, colonic stricture, fistula (eg, colocutaneous, colovaginal, and colovesical), active malignancy, earlier resection of sigmoid colon or rectum, acute diverticulitis that had not settled (elevated inflammatory markers or fever), or no colonoscopy or sigmoidoscopy or virtual colonoscopy within 2 years; if they were younger than 18 years or older than 75 years or pregnant; or if they were unable to answer the health survey. All patients gave written informed consent before randomization.

### Randomization

Patients were randomly allocated (1:1) to either elective laparoscopic sigmoid resection or conservative treatment. The allocated intervention was not blinded.

### Procedures

Patients randomized to conservative treatment received standardized written information regarding diverticulosis and constipation, which advised patients to increase the fiber content in their diet, and were prescribed a fiber supplement. Patients allocated to elective laparoscopic sigmoid resection were scheduled for surgery within 3 months of randomization. After surgery, the patients were given the same standardized written information as the patients in the conservative treatment arm.

Conservative treatment was planned to continue for at least 6 months from randomization unless an absolute indication for surgery emerged (such as fistula, stricture, or perforation). The protocol allowed patients randomized to the conservative group to undergo elective sigmoid resection after 6 months from randomization if desired. Patients were allowed to withdraw their consent to participate in the trial at any time.

### Outcomes

The primary outcome of the trial was the difference in Gastrointestinal Quality of Life Index (GIQLI) score at randomization and 6 months after randomization, and has been reported earlier along with other outcomes that were assessable within 6 months.^18^ Secondary outcomes were GIQLI at 12, 24, 48, and 96 months; Short-Form Health Survey 36 (SF-36) score at 6, 12, 24, 48, and 96 months; recurrence and severity of recurrent diverticulitis (Hinchey classification); emergency surgery due to diverticulitis; elective sigmoid resection in the patients allocated to conservative treatment; complications due to elective sigmoid resection; mortality of any cause; complications of diverticular disease; and stoma rate. Secondary outcomes up to 24-month follow-up are reported here.

Patients were contacted by mail at 12 and 24 months. In case the patients did not respond to letter or if the answers in the questionnaires were unclear, the patients were contacted by phone. Of 85 patients randomized and included, 75 and 70 were available for QOL outcomes at 1 year and 2 years, respectively, and 79 and 78 were available for the recurrence outcome at 1 year and 2 years, respectively. The data were collected prospectively using electronic case report forms. The present analysis was conducted from September 2015 to June 2022.

### Statistical Analysis

Based on sample size calculations for the primary outcome,^18^ the study aimed to recruit 133 patients. However, the trial was prematurely stopped due to significant difference in the primary outcome in the interim analysis.

Continuous outcomes that were normally distributed (GIQLI score and new cases of diverticulitis) were compared using *t* test, and effect size was reported as mean difference with 95% CI. The minimal clinically important difference for the GIQLI score has been estimated to range between 6.42 and 7.64.^20^ Continuous outcomes that were not normally distributed (physical component score and mental component score of SF-36 at 12 and 24 months)^21^ were compared using Mann-Whitney *U* test, and effect size was reported as *r* = *Z* / √*N*) without 95% CI. Imputation of the missing items (regression method) in GIQLI was performed if the questionnaire was at least 75% filled out (isolated items were missing from 3 patients at baseline, 4 patients at 12 months, and 2 patients at 24 months). Except for GIQLI, individuals with missing data were omitted from analyses. Categorical outcomes were compared using Fischer exact test (if expected cases in 1 cell <5) or χ^2^ test, and effect size was reported as odds ratio (OR) with 95% CI. All analyses were performed using SPSS version 25 (IBM). Main analyses were carried out using intention-to-treat principle, wherein patients are analyzed in the group they were randomized to instead of the treatment they actually received. Considering the high number of patients that eventually had surgery even though they were originally in the conservative treatment group, post hoc per-protocol analyses were also performed, wherein only patients who received the allocated treatment and did not crossover to the other group were included.

## Results

Between September 29, 2014, and October 10, 2018, 128 patients were assessed for eligibility, 90 of whom (28 male [31%]; mean [SD] age, 54.11 [11.9] years and 62 female [69%]; mean [SD] age, 57.13 [7.6] years) were enrolled and randomized either to surgery (n = 45) or conservative treatment (n = 45). After exclusions, a total of 85 patients were included in the intention-to-treat analysis (41 in the surgery group and 44 in the conservative group) (Figure). Baseline characteristics have been reported earlier^18^ and are shown in the eTable in Supplement 2.

**Figure.**
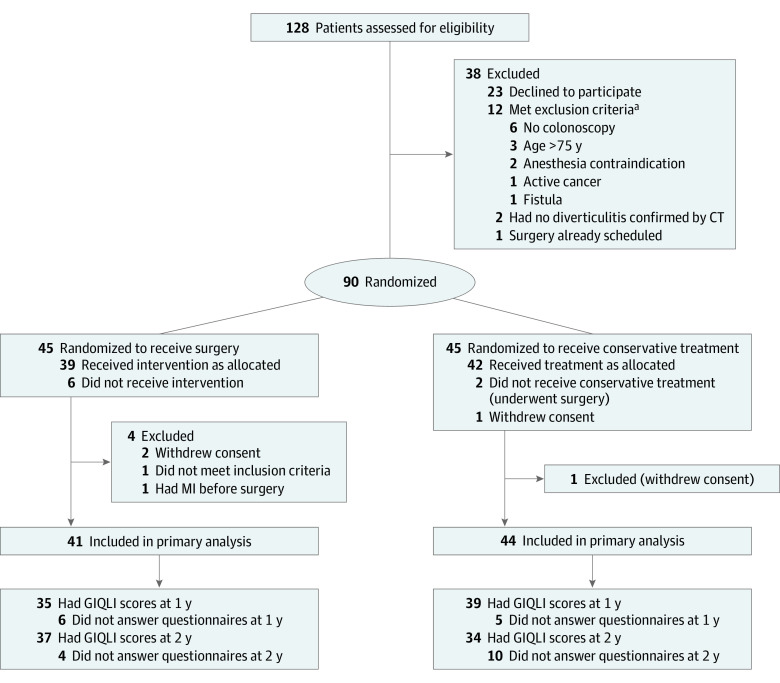
Flowchart of the Trial for 12- and 24-Month Outcomes CT indicates computed tomography; GIQLI, Gastrointestinal Quality of Life; MI, myocardial infarction. ^a^One patient met 2 exclusion criteria (no colonoscopy and anesthesia contraindication).

Four patients randomized to conservative treatment underwent elective laparoscopic sigmoid resection within 12 months (3 patients due to recurrent diverticulitis and pain and 1 due to chronic pain), and 1 patient underwent emergency open sigmoid resection due to an abscess. An additional 3 patients randomized to conservative treatment underwent elective sigmoid resection between 12 and 24 months, all due to recurrent diverticulitis and pain and 1 also due to antibiotic allergies that developed after successive antibiotic treatments. In total, 5 of 44 (11%) and 8 of 44 (18%) patients randomized to conservative treatment underwent sigmoid resection within 12 and 24 months, respectively (Table 1). Two of 41 patients (5%) randomized to surgery and included in the intention-to-treat analyses declined to undergo sigmoid resection.

**Table 1.** Operative Characteristics and Postoperative Complications (12 and 24 Months)

	No. (%)			
	At 12 mo		At 24 mo	
	Surgery group (n = 41)^a^	Conservative treatment group (n = 44)^a^	Surgery group (n = 41)	Conservative treatment group (n = 44)
Surgery				
Laparoscopy	38 (93)	4 (9)	38 (93)	7 (16)
Conversion to open surgery	1 (2)	0	1 (2)	0
Open	0	1 (2)	0	1 (2)
Stoma in primary operation	0	1 (2)	0	1 (2)
30-d Postoperative complications (Clavien-Dindo)				
I	9 (22)	1 (2)	9 (22)	0
Dermatitis	1 (2)	0	1 (2)	0
Pain	2 (5)	0	2 (5)	0
Seroma	1 (2)	0	1 (2)	0
Hematuria	1 (2)	0	1 (2)	0
Fever	1 (2)	0	1 (2)	0
Thrombophlebitis	1 (2)	0	1 (2)	0
Nausea	1 (2)	0	1 (2)	0
Superficial wound infection	1 (2)	1 (2)	1 (2)	1 (2)
II	2 (5)	0	2 (5)	0
Urinary tract infection	1 (2)	0	1 (2)	0
Anastomotic intraluminal bleeding	1 (2)	0	1 (2)	0
IIIa	2 (5)	0	2 (5)	0
Abscess (percutaneous drainage)	2 (5)	0	2 (5)	0
IIIb	2 (5)	1 (2)	2 (5)	2 (5)
Anastomotic leakage, reoperation	2 (5)	1 (2)	2 (5)	2 (5)
IV	0	0	0	0
Late complications, incisional hernia	0	0	1 (2)	1 (2)

^a^ Forty-five patients were randomized to surgery, 41 were included in the analyses, and 39 received the allocated intervention (surgery). Forty-five patients were randomized to conservative treatment, 44 were included in the analyses, and 42 received the allocated intervention (conservative treatment and no surgery within 6 months).

Eleven of 41 patients (27%) randomized to surgery had minor postoperative complications and 4 of 41 patients (10%) had major postoperative complications (Clavien-Dindo III or higher) (Table 1). One of 44 patients (2%) randomized to conservative treatment had minor postoperative complications and 2 of 44 (5%) had major postoperative complications within 24 months from randomization (Table 1). One patient in each group developed an incisional hernia within 24 months and both underwent hernia repair. Two patients randomized to surgery and 3 patients randomized to conservative treatment had temporary stoma. All but 1 patient in the conservative treatment group had undergone successful stoma reversal surgery by 24 months. There was no mortality within 24 months from randomization.

Thirty-five of 41 (85.4%) and 37 of 41 (90.2%) patients randomized to surgery and 39 of 40 (88.6%) and 34 of 44 (77.3%) patients randomized to conservative treatment answered QOL questionnaires at 12 and 24 months, respectively. Mean GIQLI score at 12 months was 9.51 points higher in the surgery group compared to the conservative treatment group (mean [SD], 118.54 [17.95] vs 109.03 [19.32]; 95% CI, 0.83-18.18; *P* = .03), while mean the GIQLI score at 24 months was similar between the groups (Table 2). The mean (SD) mental component score of SF-36 was higher in the surgery group compared to the conservative treatment group at 12 months (55.89 [12.96] vs 54.05 [12.02]; *P* = .047) but not at 24 months (Table 2). The physical component score of SF-36 was similar between the groups at 12 and 24 months (Table 2).

**Table 2.** Outcomes Within 12 and 24 Months

	No. (%)		*P* value	Effect size^a^
	Surgery (n = 41)	Conservative treatment (n = 44)		
**GIQLI score, mean (SD)**				
At 12 mo^b^	118.54 (17.95)	109.03 (19.32)	.03	9.51 (0.83 to 18.18)
At 24 mo^c^	116.34 (15.56)	108.85 (18.88)	.07	7.49 (−0.675 to 15.65)
**SF-36 score, median (IQR)**				
At 12 mo^d^				
PCS	51.91 (13.24)	51.21 (11.50)	.25	0.14^e^
MCS	55.89 (12.96)	54.05 (12.02)	.047	0.23^e^
At 24 mo^f^				
PCS	49.37 (11.58)	49.64 (11.75)	.13	0.18^e^
MCS	54.49 (9.84)	54.42 (11.58)	.53	0.08^e^
**Patients with recurrent episodes of diverticulitis**				
Within 12 mo^g^				
Any	3 (8)	21 (51)	<.001	12.25 (3.24 to 46.25)
Hinchey stage 0 or Ia	3 (8)	20 (49)		
Hinchey stage Ib	0	1 (2)		
Hinchey stage II	0	0		
Hinchey stage III	0	0		
Hinchey stage IV	0	0		
Within 24 mo^h^				
Any	4 (11)	25 (61)	<.001	12.89 (3.84 to 43.34)
Hinchey stage 0 or Ia	4 (11)	24 (59)		
Hinchey stage Ib	0	1 (2)		
Hinchey stage II	0	0		
Hinchey stage III	0	0		
Hinchey stage IV	0	0		
**Stoma**				
Within 12 mo				
Permanent	0	0	NA	NA
Temporary	2 (5)	2 (5)		
Reversal within 12 mo	2 (100)	1 (50)	NA	NA
Within 24 mo				
Permanent	0	0	NA	NA
Temporary	2 (5)	3 (7)		
Reversal within 24 mo	2 (100)	2 (67)	NA	NA
**Mortality**				
Within 24 mo	0	0	NA	NA

Abbreviations: GIQLI, Gastrointestinal Quality of Life; MCS, mental component score; NA, not applicable; PCS, physical component score; SF-36, Short-Form Health Survey 36.

^a^ Effect size is mean difference (95% CI) in continuous variables and odds ratio (95% CI) in categorical variables.

^b^ Data missing for 6 patients in the surgery group and 4 patients in the conservative group.

^c^ Data missing for 5 patients in the surgery group and 10 patients in the conservative group.

^d^ Data missing for 7 patients in the surgery group and 6 patients in the conservative group.

^e^ Effect size is here given as *r* = Z / *√*N without 95% CI.

^f^ Data missing for 5 patients in the surgery group and 13 patients in the conservative group.

^g^ Data missing for 3 patients in the surgery group and 3 patients in the conservative group.

^h^ Data missing for 4 patients in the surgery group and 3 patients in the conservative group.

Within 12 months from randomization, 21 patients in the conservative treatment group had recurrent diverticulitis compared to 3 patients in the surgery group (Table 2). Within 24 months, 25 patients in the conservative treatment group had recurrent diverticulitis (including 1 with complicated diverticulitis with abscess requiring emergency surgery) compared to 4 patients in the surgery group (Table 2). One of 4 patients with recurrent diverticulitis in the surgery group had not undergone surgery.

At 12 months, patients in both groups were similarly satisfied with the treatment, although pain was reported to be more frequent and intensive in the conservative group compared to the surgery group (Table 3). At 24 months, patients in the surgery group were more satisfied and reported significantly less frequent and less intensive pain (Table 3).

**Table 3.** Patients’ Perceptions and Pain at 12 and 24 Months From Randomization

	No. (%)		*P* value
	Surgery	Conservative treatment	
**Patient satisfaction with assigned treatment**			
At 12 mo^a^			
Satisfied	31 (100)	33 (89)	.17
Not satisfied	0	2 (5)	
Could not tell	0	2 (5)	
At 24 mo^b^			
Satisfied	34 (97)	27 (79)	.03
Not satisfied	1 (3)	1 (3)	
Could not tell	0	6 (18)	
**Pain**			
At 12 mo^c^			
No pain	19 (58)	12 (32)	.04
Once a month	6 (18)	12 (32)	
Once a week	5 (15)	9 (24)	
Few times a week	1 (3)	3 (8)	
Every day	2 (6)	1 (3)	
Several times a day	0	0	
All the time	0	0	
At 24 mo^d^			
No pain	18 (51)	7 (20)	.006
Once a month	8 (23)	13 (37)	
Once a week	8 (23)	8 (23)	
Few times a week	0	4 (11)	
Every day	1 (3)	2 (6)	
Several times a day	0	0	
All the time	0	1 (3)	
**Pain (VAS score), mean (SD)**			
At 12 mo^e^	1.32 (1.76)	2.64 (2.14)	.008
At 24 mo^f^	1.59 (1.55)	3.18 (2.38)	.001

Abbreviation: VAS, visual analog scale.

^a^ Responses received for 31 patients in the surgery group and 37 in the conservative group.

^b^ Responses received for 35 patients in the surgery group and 34 in the conservative group.

^c^ Responses received for 33 patients in the surgery group and 37 in the conservative group.

^d^ Responses received for 35 patients in the surgery group and 35 in the conservative group.

^e^ Responses received for 31 patients in the surgery group and 36 in the conservative group.

^f^ Responses received for 37 patients in the surgery group and 34 in the conservative group.

The post hoc per-protocol analysis included 39 patients that were randomized to surgery and underwent surgery and 39 patients (for 12-month outcomes) or 36 patients (for 24-month outcomes) who were randomized to conservative treatment and did not undergo surgery within 12 months or 24 months, respectively. Mean GIQLI score was 11.27 points higher in the surgery group compared to the conservative treatment group at 12 months (mean [SD], 119.42 [17.98] vs 108.15 [19.28]; 95% CI, 2.24-0.29; *P* = .02) and 10.43 points higher at 24 months (mean [SD], 117.24 [15.51] vs 106.82 [18.94]; 95% CI, 1.52-19.33; *P* = .02) (Table 4). At 12 months, the median (IQR) mental component score of SF-36 was higher in the surgery group compared to conservative treatment group (55.37 [12.47] vs 50.45 [16.07]; *P* = .048), but the physical component score of SF-36 was similar between groups (Table 4). At 24 months, there was no difference in either physical component score or mental component score between the groups.

**Table 4.** Per-Protocol Analyses at 12 and 24 Months

	No. (%)		*P* value	Effect size^a^
	Surgery (n = 39)	Conservative treatment (n = 39 at 12 mo and 36 at 24 mo		
**GIQLI score, mean (SD)**				
At 12 mo^b^	119.42 (17.98)	108.15 (19.28)	.02	11.27 (2.24-20.29)
At 24 mo^c^	117.24 (15.51)	106.82 (18.94)	.02	10.43 (1.52-19.33)
**SF-36 score, median (IQR)**				
At 12 mo^d^				
PCS	53.06 (13.40)	50.14 (11.44)	.19	0.16^e^
MCS	55.37 (12.47)	50.45 (16.07)	.048	0.24^e^
At 24 mo^f^				
PCS	50.63 (11.10)	48.95 (10.21)	.14	0.19^e^
MCS	54.91 (9.70)	54.42 (9.86)	.55	0.08^e^
**Patients with recurrent episodes of diverticulitis**				
Within 12 mo^g^				
Any	3 (8)	16 (44)	.001	8.80 (2.27-34.03)
Hinchey stage 0 or Ia	3 (8)	16 (44)		
Hinchey stage Ib	0	0		
Hinchey stage II	0	0		
Hinchey stage III	0	0		
Hinchey stage IV	0	0		
Within 24 mo^h^				
Any	3 (9)	18 (55)	<.001	12.80 (3.26-50.25)
Hinchey stage 0 or Ia	3 (9)	18 (55)		
Hinchey stage Ib	0	0		
Hinchey stage II	0	0		
Hinchey stage III	0	0		
Hinchey stage IV	0	0		

Abbreviations: GIQLI, Gastrointestinal Quality of Life; MCS, mental component score; PCS, physical component score; SF-36, Short-Form Health Survey 36.

^a^ Effect size is mean difference (95% CI) in continuous variables and odds ratio (95% CI) in categorical variables.

^b^ Data missing for 5 patients in the surgery group and 3 patients in the conservative group.

^c^ Data missing for 4 patients in the surgery group and 8 patients in the conservative group.

^d^ Data missing for 6 patients in the surgery group and 5 patients in the conservative group.

^e^ Effect size is here given as *r* = Z / *√*N without 95% CI

^f^ Data missing for 5 patients in the surgery group and 10 patients in the conservative group.

^g^ Data missing for 3 patients in the surgery group and 3 patients in the conservative group.

^h^ Data missing for 4 patients in the surgery group and 3 patients in the conservative group.

QOL of patients who were randomized to conservative treatment but underwent sigmoid resection during follow-up was analyzed post hoc. Among patients randomized to conservative treatment, the mean (SD) baseline (at randomization) GIQLI score was 106.75 (18.59) for patients who remained in conservative treatment vs 91.38 (20.98) for patients who underwent surgery in the follow-up period (mean difference, 15.38; 95% CI, −0.61 to 31.36; *P* = .06). In an effort to compare QOL in these patients before crossing over to surgery, preoperative GIQLI score was compared to 2-year GIQLI scores of patients continuing conservative treatment. The mean (SD) GIQLI score at 2 years was 106.20 (19.46) for patients who remained in conservative treatment vs the mean preoperative GIQLI score of 82.38 (26.16) for patients who were randomized to conservative treatment but eventually underwent surgery within 2 years (mean difference, 23.82; 95% CI, 6.44 to 41.21; *P* = .009).

## Discussion

In this prespecified 2-year follow-up of a randomized clinical trial, we randomly assigned patients with recurrent, complicated, or persistent painful diverticulitis to receive either elective laparoscopic sigmoid resection or conservative treatment with fiber supplementation and diet modification. While significant improvement in QOL was noted at 6 months^18^ and 1 year after randomization in the surgery group, no difference was noted in QOL at 2 years between patients randomized to surgery or conservative treatment in the intention-to-treat analyses. However, surgery was highly effective in preventing acute diverticulitis episodes, as 4 of 37 patients (11%) in the surgery group had a recurrent episode compared to 25 of 41 (61%) in the conservative group by 24 months. Of note, one-fourth of the patients having recurrences after being randomized to surgery had not actually undergone surgery. Further, more patients in the surgery group were satisfied with the assigned treatment and reported less frequent and less severe pain. Possibly because of the high frequency of recurrent episodes of diverticulitis, 11% of patients randomized to conservative treatment had already undergone sigmoid resection by 12 months. Within 2 years, 18% of patients in conservative group had had surgery. Because of the high rate of crossover between the groups in the follow-up, we carried out per-protocol analyses. These analyses demonstrated higher QOL in patients who were randomized to and underwent surgery compared to patients who were randomized to and remained in conservative treatment. While the benefits of surgery need to be weighed against possible risk of postoperative complications, it must be noted that 5% of patients randomized to conservative treatment experienced major postoperative complications (compared to 10% in the surgery group) and 7% needed a stoma (compared to 5% in the surgery group). One patient in each group developed an incisional hernia in the follow-up.

Although several retrospective or nonrandomized studies have pointed toward improved QOL after elective sigmoid resection for recurrent diverticulitis,^22,23^ only 1 earlier randomized clinical trial comparing surgery to conservative treatment exists to our knowledge. The Dutch DIRECT trial, which is highly similar to the current LASER trial, recently published results of their 5-year follow-up.^17^ Although the DIRECT trial does not report outcomes at 2 years, 1-year results of the trials can be compared. While the raw GIQLI scores differ slightly (surgery group: DIRECT, 112.8 vs LASER, 118.5 and conservative group: DIRECT, 101.2 vs LASER, 109.0), the difference between the groups at 1 year is almost the same in these 2 trials (11.6 in DIRECT; 9.5 in LASER) both favoring surgery in terms of QOL. A considerably higher number of patients randomized to conservative treatment underwent surgery during follow-up in the DIRECT trial (23% at 6 months and 46% at 5 years) than in the LASER trial (4% at 6 months and 18% at 2 years).^18^ There were also slightly more major postoperative complications in the surgery group in the DIRECT trial (21%) compared to the LASER trial (10%), while postoperative complication rates in conservative groups were roughly similar across the trials at 6 months (2% and 5%, respectively). The protective stoma rate was also slightly higher in the surgery group of the DIRECT trial (19%) compared to the LASER trial (5%). There was only a 2% incisional hernia rate in the LASER trial within 2-year follow-up, while the DIRECT trial reported a 17% incisional hernia rate in the surgery group at 5-year follow-up. While the recurrence rate of diverticulitis in the surgery group was similar between the trials (11% in both), 61% patients in the conservative group in the LASER trial had already experienced recurrence within 2 years compared to a 30% recurrence rate within 5 years in the DIRECT trial. This difference might reflect the slightly different inclusion criteria used in the trials. While 78% of patients in the LASER trial were included based on recurrent diverticulitis, 63% of patients in the DIRECT trial were recruited due to persistent pain.

### Limitations

The LASER trial has limitations. First, the trial was prematurely terminated for benefit. However, this was done in accordance with the prespecified criteria in the original study protocol. Second, partly due to the premature termination, the study sample is relatively small. It must be noted though that the trial had run for 4 years before termination and recruiting to a trial comparing surgery with conservative treatment is extremely challenging. The DIRECT trial was also prematurely stopped because of problems with recruitment.^16^ Third, the study population was slightly heterogenous, as patients with a complicated or persistent painful diverticulitis were included in addition to the main group of patients, those with recurrent diverticulitis. The small number of patients with other than recurrent diverticulitis prevented subgroups analyses. Fourth, 9% to 22% of patients (depending on the time point and group) did not return QOL questionnaires, which could have introduced bias. On the other hand, questionnaire response rates are never 100%, and a more-than 80% response rate can be considered high. Fifth, one-fifth of patients randomized to conservative treatment underwent sigmoid resection within 2 years. While this may have influenced the outcomes of the conservative group, we carried out per-protocol analyses to mitigate the effect. The observed improvement in QOL in the surgery group was more pronounced in these analyses. The small number of patients crossing over from the conservative group to surgery (8 patients) and the short follow-up after surgery prevented us from analyzing them and the implications of the delayed surgery in more detail. Sixth, a placebo effect with surgery cannot be ruled out completely. Seventh, the current study reports outcomes within 2 years from randomization and outcomes may change in longer follow-up. We will report outcomes at 4 and 8 years when they are available.

## Conclusions

The results of the current study have implications for clinical practice. Elective sigmoid resection increased QOL and reduced future recurrences of diverticulitis of patients with either recurrent or persistent painful diverticulitis in both randomized clinical trials on the topic. These benefits must be weighed against possible harms of surgery. As can be noted from the relatively high rate of crossing over from the conservative group to surgery in both trials, many patients with symptoms or recurrences chose surgery over conservative treatment, and the potential for harm with surgery in these patients initially undergoing conservative treatment is merely postponed. Several current guidelines suggest an individualized case-by-case selection approach to elective sigmoid resection in patients with recurrent diverticulitis without clearly defining indications when such should be offered.^12,13,14,15^ The risk of further episodes of diverticulitis can be predicted and is largely based on the number of earlier episodes of diverticulitis.^24^ While patients with first-time uncomplicated diverticulitis have only a 30% risk of recurrence within 5 years, patients who have had 3 or more earlier cases of diverticulitis have about an 80% chance of another recurrence.^24^ Decisions to proceed to elective sigmoid resection for recurrent or painful diverticulitis needs to be made together with the patient using shared decision-making and considering the benefits and harms of both surgery and conservative treatment. Three or more episodes of diverticulitis seemed to serve as an appropriate threshold for offering surgical options in this study.

## Notes

Supplement 1. Trial protocol

Supplement 2. eTable 1. Baseline characteristics

Supplement 3. Data sharing statement
