# Supplementary material for: Quality-of-Life and Recurrence Outcomes Following Laparoscopic Elective Sigmoid Resection vs Conservative Treatment Following Diverticulitis: Prespecified 2-Year Analysis of the LASER Randomized Clinical Trial
Source: JAMA Surg. 2023 Apr 19;158(6):593–601. doi: 10.1001/jamasurg.2023.0466 (PMC10116381; doi:10.1001/jamasurg.2023.0466)
Supplement: Supplement 2. — eTable 1. Baseline characteristics [file jamasurg-e230466-s002.pdf]

Supplemental Online Content

Santos A, Mentula P, Pinta T, et al. Quality-of-life and recurrence outcomes following laparoscopic elective sigmoid resection vs conservative treatment following diverticulitis: prespecified 2-year analysis of the LASER randomized clinical trial. *JAMA Surg*. Published online April 19, 2023. doi:10.1001/jamasurg.2023.0466

eTable. Baseline characteristics

This supplemental material has been provided by the authors to give readers additional information about their work.

© 2023 Santos A et al. *JAMA Surgery.*

eTable 1. Baseline characteristics

	**Surgery** (N = 41)	**Conservative treatment** (N = 44)
Age, years - median (IQR)	59 (51.5-63)	59 (50.3-62.9)
**Sex**, male - no. (%)	11 (27%)	15 (34%)
**Body Mass Index**, kg/m^2 - mean (SD)	29.3 (4.72)	28.7 (4.15)
**Comorbidities - no. (%)**		
Coronary disease/Myocardial infarction	0	1 (2%)
Congestive heart failure	0	1 (2%)
Atrial fibrillation	1 (2%)	1 (2%)
Hypertension	15(35%)	11 (26%)
Peripheral vascular disease	0	1 (2%)
Cerebrovascular disease	0	0
Hemiplegia	0	0
Dementia	0	0
COPD or asthma	3 (7%)	4 (10%)
Connective tissue disease	3 (7%)	3 (7%)
Liver disease	0	0
Peptic ulcer	0	0
Diabetes mellitus	1 (2%)	5 (12%)
without complications	1 (2%)	4 (10%)
with complications	0	1 (2%)
Kidney disease (moderate / severe)	0	0
Cancer	0	0
Leukemia	0	0
Lymphoma	0	0
AIDS	0	0
**Inclusion criteria – no. (%)#**		
Recurrent diverticulitis	34 (82%)	32 (77%)
Complicated diverticulitis##	10 (24%)	13 (31%)
Persistent pain >3 months after diverticulitis	2 (5%)	3 (7%)
**Frequency of pain at randomisation – no. (%)**	4 (10%)	3 (7%)
never	12 (29%)	7 (16%)
once a month	7 (17%)	8 (18%)
once a week	8 (20%)	9 (21%)
few times a week	1 (2%)	5 (11%)
everyday	0	1 (2%)
several times a day	2 (5%)	1 (2%)
all the time		
**Pain, mean VAS (SD)**	4.2 (2.9)	4.6 (2.6)
**GIQLI at randomisation**	102.5 (21.64)	101.83 (19.4)
**SF-36 at randomisation median**		
PCS	47.71 (11.28)	43.40 (12.99)
MCS	54.56 (15.52)	49.36 (17.21)
**Mean episodes of diverticulitis (SD)**	4.6 (3.5)	4.0 (3.1)
**Most severe diverticulitis before randomisation – no. (%)**	7 (17%)	11 (25%)
Hinchey 0	23 (56%)	19 (43%)
Hinchey Ia	5 (12%)	8 (18%)
Hinchey Ib	3 (7%)	5 (11%)
Hinchey II	2 (2%)	0
Hinchey III		

© 2023 Santos A et al. *JAMA Surgery.*

**Most invasive treatment for earlier diverticulitis – no. (%)**	1 (2%)	1 (2%)
Symptomatic treatment	30 (73%)	29 (66%)
Antibiotics	6 (15%)	9 (21%)
Percutaneous drainage	2 (5%)	1 (2%)
Laparoscopic lavage		
**Location of diverticulosis – no. (%)**		
whole colon	3 (7%)	5 (11%)
sigmoid	24 (58%)	24 (55%)
sigmoid and transverse	5 (12%)	1 (2%)
descending colon	9 (22%)	14 (32%)
**Earlier treatment of diverticulosis* – no. (%)**	11 (27%)	10 (23%)
No treatment	23 (56%)	25 (57%)
Repeated per oral antibiotics	22 (54%)	24 (55%)
Fibre supplement	18 (44%)	14 (32%)
Behavioural†		
**Medication, no. (%)**		
Anticoagulative medication	3 (7%)	1 (2%)
Corticosteroid medication	1 (2%)	1 (2%)
Immunosuppressive medication	3 (7%)	3 (7%)

No significant differences were identified between the treatment groups in any baseline variables. The table has been published in the original article[17]. Abbreviations : COPD – Chronic Obstructive Pulmonary Disease, AIDS – Acquired Immunodeficiency Syndrome, VAS – Visual Analog Scale, GIQLI – Gastrointestinal Quality of Life, SF-36 – Short Form 36, PCS – Physical component score, MCS – Mental component score

#One patient could met more than one inclusion criteria

##Complicated diverticulitis were abscesses

*More than one form of treatment could be assigned per patient.

†Diet changes in order to accommodate higher levels of fibre intake. Treatment of constipation if necessary.

© 2023 Santos A et al. *JAMA Surgery.*
