# Supplementary material for: Quality-of-Life and Recurrence Outcomes Following Laparoscopic Elective Sigmoid Resection vs Conservative Treatment Following Diverticulitis: Prespecified 2-Year Analysis of the LASER Randomized Clinical Trial
Source: JAMA Surg. 2023 Apr 19;158(6):593–601. doi: 10.1001/jamasurg.2023.0466 (PMC10116381; doi:10.1001/jamasurg.2023.0466)
Supplement: Supplement 3. — Data sharing statement [file jamasurg-e230466-s003.pdf]

Data Sharing Statement

Santos. Quality-of-Life and Recurrence Outcomes Following Laparoscopic Elective Sigmoid Resection vs Conservative Treatment Following Diverticulitis. *JAMA Surg*. Published April 19, 2023. doi:10.1001/jamasurg.2023.0466

Data
Data available: No

Additional Information
Explanation for why data not available: Study permits do not allow sharing individual patient data.
